# Ultrafast Excited State Dynamics in a First Generation Photomolecular Motor

**DOI:** 10.1002/cphc.201901179

**Published:** 2020-02-03

**Authors:** Andy S. Sardjan, Palas Roy, Wojciech Danowski, Giovanni Bressan, Laura Nunes dos Santos Comprido, Wesley R. Browne, Ben. L. Feringa, Stephen R. Meech

**Affiliations:** ^1^ Molecular Inorganic Chemistry Stratingh Institute for Chemistry University of Groningen Nijenborgh 4 9747AG Groningen The Netherlands; ^2^ School of Chemistry University of East Anglia Norwich Research Park Norwich NR4 7TJ UK; ^3^ Synthetic Organic Chemistry Stratingh Institute for Chemistry University of Groningen Nijenborgh 4 9747AG Groningen The Netherlands

**Keywords:** excited state, fluorescence, coherence, molecular motor, photochemistry, ultrafast dynamics

## Abstract

Efficient photomolecular motors will be critical elements in the design and development of molecular machines. Optimisation of the quantum yield for photoisomerisation requires a detailed understanding of molecular dynamics in the excited electronic state. Here we probe the primary photophysical processes in the archetypal first generation photomolecular motor, with sub‐50 fs time resolved fluorescence spectroscopy. A bimodal relaxation is observed with a 100 fs relaxation of the Franck‐Condon state to populate a red‐shifted state with a reduced transition moment, which then undergoes multi‐exponential decay on a picosecond timescale. Oscillations due to the excitation of vibrational coherences in the S_1_ state are seen to survive the ultrafast structural relaxation. The picosecond relaxation reveals a strong solvent friction effect which is thus ascribed to torsion about the C−C axle. This behaviour is contrasted with second generation photomolecular motors; the principal differences are explained by the existence of a barrier on the excited state surface in the case of the first‐generation motors which is absent in the second generation. These results will help to provide a basis for designing more efficient molecular motors in the future.

## Graphical abstract

**Generation conflict?** The excited state dynamics of first‐generation molecular motors are studied by means of ultrafast time resolved fluorescence. A 100 fs structural reorganization in the excited state is followed by a slower excited state relaxation, which is influenced by solvent viscosity. The decay is accompanied by coherent vibrational excitation of modes in the excited electronic state. The observed behaviour is contrasted with that of the second‐generation motors.

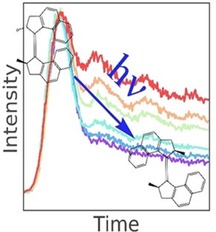

## 1 Introduction

**Scheme 1:**
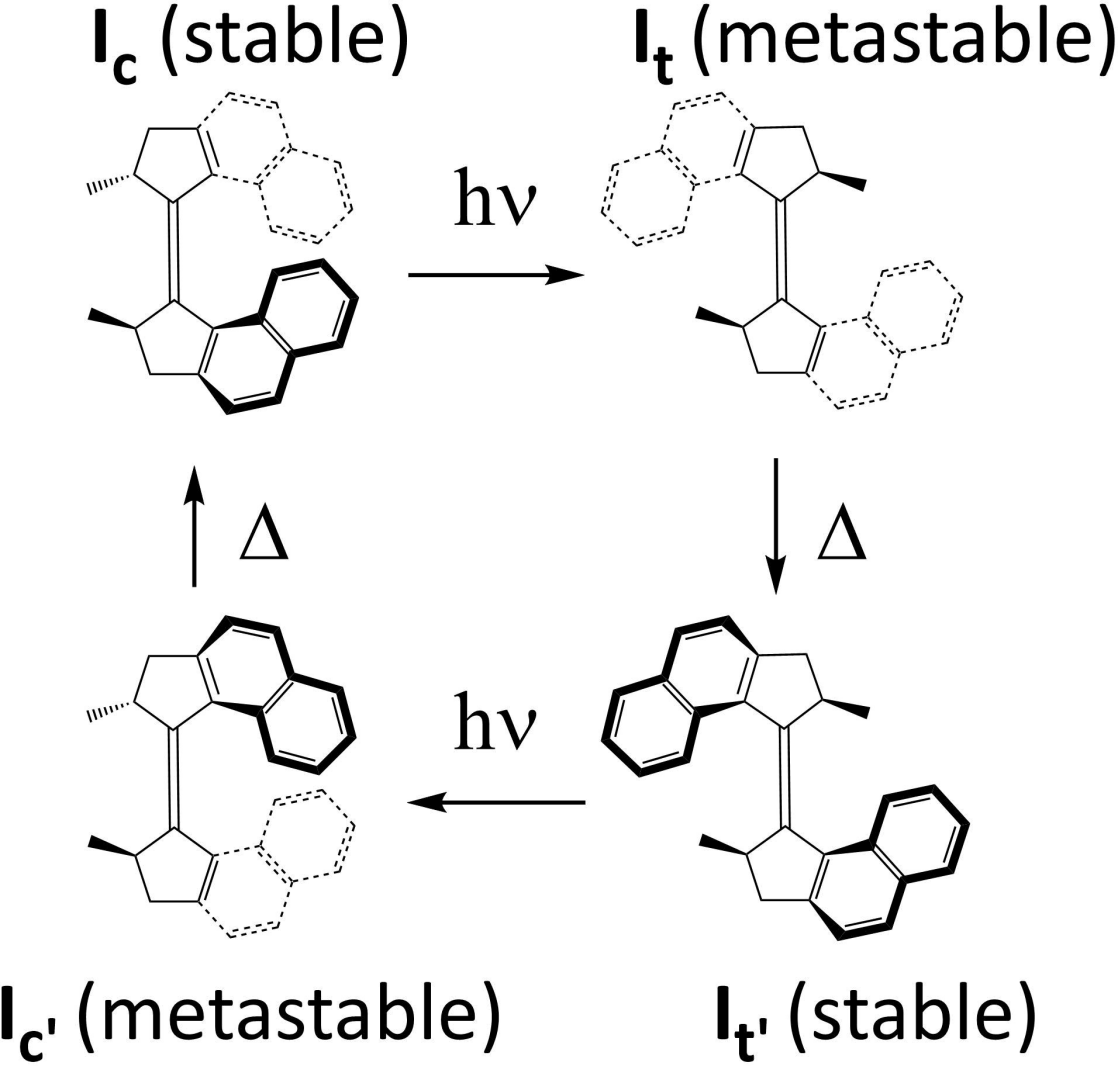
Photocycle for the first‐generation motor. The **I**_c_ to **I**_t_ photochemical step is the focus of this study.

Recent decades have seen rapid progress in the development of light driven nanomolecular machines.^1^ Among the most promising power sources for such machines are the overcrowded‐alkene based photomolecular motors.1b, 1d–1f, 2 The first generation of such motors, which are based on an overcrowded alkene geometry with two stereogenic centres, undergo a unidirectional rotation via four steps, comprising of successive photochemical isomerisation and thermal helix inversion reactions (Scheme 1).1d, 3 These first generation motors showed high quantum yields for photochemical isomerisation and a favourable position of the photostationary state.3b However, the relatively high barrier in the rate determining thermal helix inversion step restricted the accessible rotation rate. This prompted the development of second‐generation motors, in which one half was replaced by an extended aromatic group (e. g. fluorene, xanthene).^4^ Second generation motors follow the same basic four‐step mechanism of Scheme 1, but require only a single stereogenic centre. Some derivatives of this structure showed greatly accelerated rates of thermal helix inversion, with up to MHz rates of rotation.^5^ However, in general second generation motors exhibit lower quantum yields and less favourable positions of the photostationary state.^6^ Clearly, it is advantageous to combine the favourable photochemical properties of first generation motors with the rapid rotation of the second. This requires optimisation of the photochemistry, which in turn entails the development of a detailed understanding of excited state processes.

Herein we present a study of the stable cis (**I**_c_) to metastable trans (**I**_t_) isomerization of the archetypal first generation motor, dimethyl tetrahydro‐bi(cyclopenta[*α*]napthal‐enylidene) (**I**, Scheme 1) by means of sub‐50 fs resolution time resolved fluorescence spectroscopy, employing solvent friction as a probe of the reaction coordinate. These data contrast with our earlier studies of the second generation motor,^6^, ^7^ and several important differences are resolved. The present work follows a recent multipulse transient absorption study of **I** in all four ground states in heptane and 2‐butanol, by Sension and co‐workers.^8^ They found that the initially excited bright state of stable **I**_c_ decays rapidly (sub‐ps) to a second bright state, that subsequently decays on the picosecond time scale to a dark state, which finally populates the metastable **I**_t_ ground state. Time resolved fluorescence of **I**_c_ complements these data by resolving the ultrafast (ca 100 fs) decay of the Franck‐Condon (FC) state, revealing the role of excited state coherences and extracting the complex spectral and temporal kinetics on the excited state surface. Further, the solvent friction dependence is measured and reports on the change in molecular shape along the reaction coordinate.

## Experimental Section

The synthesis of **I**_c_ and the apparatus for ultrafast fluorescence up‐conversion have been described in detail elsewhere.3a, 9 The concentration of **I**_c_ was adjusted to give an optical density of 0.5 in a 1 mm pathlength flow cell and 250 mL of solution was flowed through the cell at a rate of 0.5 mL s^−1^. An important consideration is that **I**_c_ converts efficiently to **I**_t_ and then on to the stable trans form. The total solution volume was sufficient to ensure that build‐up of the stable trans form was negligible, and in any case that form will not be excited by the 400 nm excitation wavelength employed. A further concern is the possibility of generating and then re‐exciting **I**_t_ in the excitation beam, which may then contribute to the measured fluorescence. This was checked both by ensuring that the replacement rate of the sample in the excited volume exceeded the conversion rate, and also by limiting the excitation power to ≤5 mW. It was further established that the measured up‐conversion intensity was linearly related to excitation power in this range (see Supplementary Information S1 for details).

In some respects measuring the steady state emission is more challenging, again due to problems with conversion to and emission from **I**_t_. Here this was avoided by flowing the samples and measuring emission using a pulsed excitation source with gated CCD detection, as described in the Supplementary Information S1.

## 2 Results

**Figure 1:**
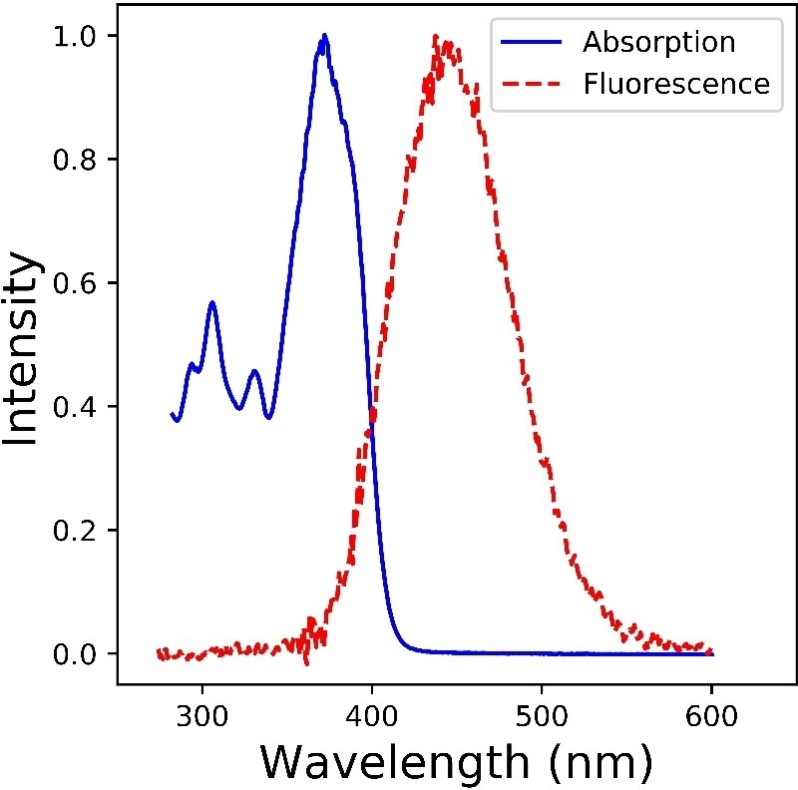
Normalised absorption (blue) and emission (red) spectra for **I**_c_ in ethanol obtained with a low repetition rate pulsed source. The emission spectrum (excitation at 355 nm) was collected as described in the Supporting Information.

Figure 1 shows steady state absorption and fluorescence spectra of **I**_c_. The absorption is narrow and slightly structured while fluorescence is red‐shifted, unstructured and broadened, consistent with a change in structure between ground and emissive states. This is expected based on the structure of **I**_c_, as excitation is localised on the bridging bond, reducing bond order and releasing steric strain between the two halves.^10^ Although the emission is weak (on the order of the solvent Raman scattering) it is readily measured and increases as the viscosity of the solvent is increased (see below). This result suggests a radiationless decay coordinate that displaces significant volumes of solvent, and is thus opposed by solvent friction.^11^

**Figure 2:**
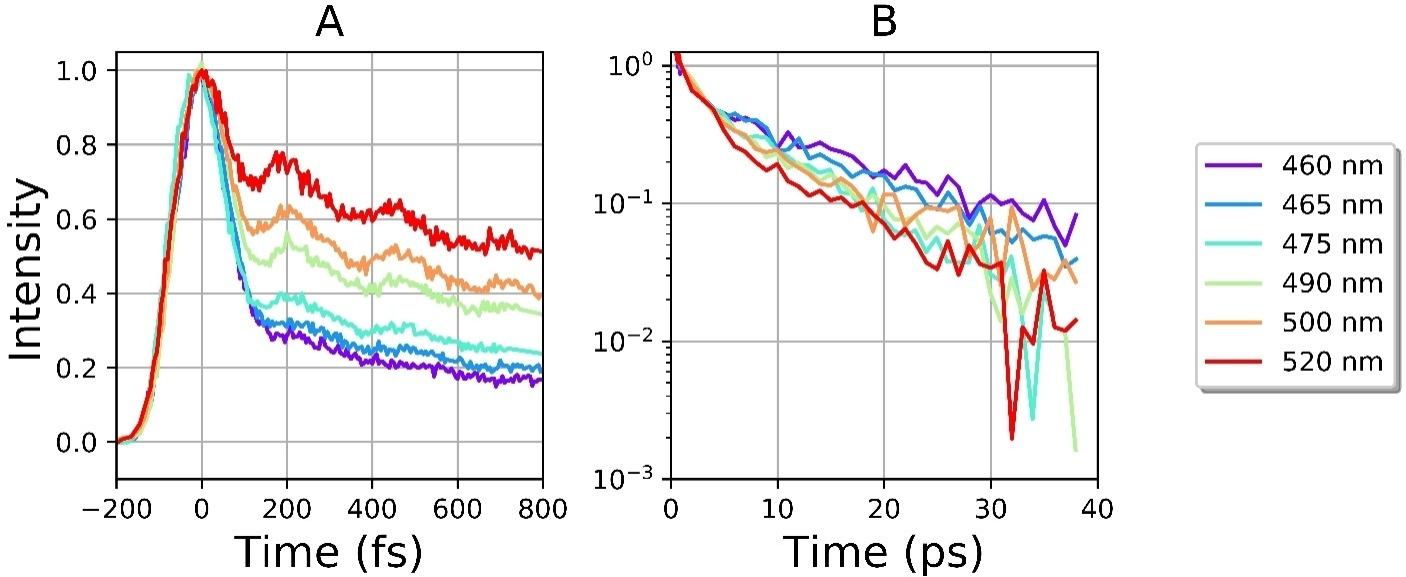
Time resolved fluorescence of **I**_c_ in ethanol as a function of emission wavelength. A) Early time response, highlighting the dominant 100 fs ultrafast decay on the blue side of the emission and coherently excited oscillations. B) Long‐time response plotted on a logarithmic intensity scale to highlight the wavelength dependence.

Figure 2 shows time resolved fluorescence of **I**_c_ in ethanol, measured as a function of the emission wavelength. There are a number of notable features. For data measured around the maximum of the emission (ca. 450 nm) an ultrafast decay (ca 100 fs) is the dominant feature (Figure 2A). However, the decay is to a weaker longer‐lived emission, indicating that the population remains on the excited state surface after the 100 fs step. The decreased amplitude is indicative of population of a state with a reduced transition moment for fluorescence, consistent with the earlier transient absorption study.^8^ The amplitude of the ultrafast component decreases as the observation wavelength is moved to the red side of the emission, although no rise time is detected at any of the wavelengths probed (see Figure 1). The slower fluorescence is itself non‐single exponential, and well fit by a biexponential function with decay components of a ca. 1 ps and a few tens of picoseconds. The mean lifetime (dominated by the longer component) decreases at longer wavelength (Figure 2B). The wavelength dependent biexponential fit data are collected in Supplementary Information S2.

**Figure 3:**
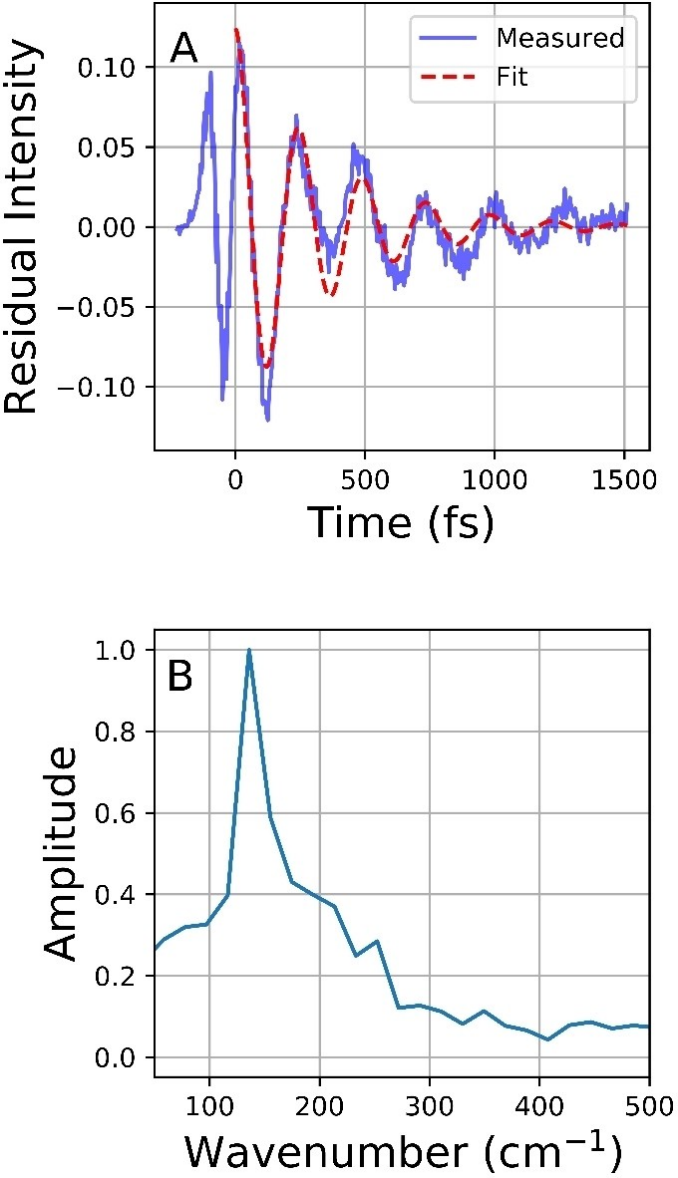
A) Residual plot following subtraction of a sum of exponentials from data sets of the type shown in Figure 2A. Also shown (red) is the fit to a single damped sine function. While the dominant frequency is well reproduced the overall fit is not good, indicating contributions from additional frequencies. B) The spectrum resulting from a Fourier transform of the data in Figure 2A. Data at around 50 fs were not included as they produced nonphysical high frequencies, probably associated with the inadequate fit of the exponential function convolved with a Gaussian to the measured data at early times.

Superimposed on the longer fluorescence decay is a coherent oscillation, which must arise from a coherently excited vibrational mode in the fluorescent state. The amplitude of modulation is greater on the red edge of the emission than at the peak (Figure 2A). Such a wavelength dependence is characteristic of a mode that modulates the energy gap between ground and excited electronic states, rather than the transition dipole moment.^12^ To further analyse the oscillatory response a data set with high signal‐to‐noise at a wavelength with significant amplitude for the oscillation was fit to a sum of exponentials. The exponential fit was subtracted and the Fourier transform of the residuals (Figure 3A) yields the spectrum in Figure 3B. The spectrum reveals a dominant mode centered at 138 cm^−1^ on top of a complex line shape indicating components at higher frequencies. In figure 3A the residuals are fit to a damped sine function with frequency (136×*c*) s^‐1^ which recovers with a damping time constant of 350 fs.

**Figure 4:**
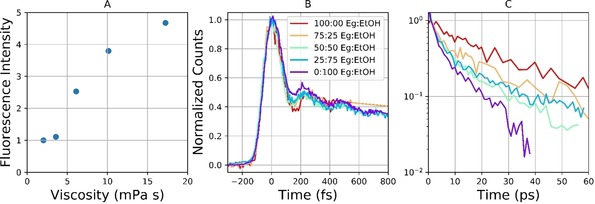
Viscosity dependent fluorescence of **I**_c_ in EtOH: Eg mixtures. A) Steady state emission intensity plotted as a function of the viscosity of the different EtOH:Eg compositions (see also Figure S1). B) Sub‐picosecond fluorescence decay as a function of Vol fraction EG shown. C) Picosecond fluorescence decay for the same solutions as in B).

Figure 4 reports the effect of solvent viscosity on the emission intensity and excited state dynamics, where viscosity is varied through the composition of an ethanol – ethylene glycol mixture. Use of this mixed solvent avoids large changes in either specific intermolecular interactions or polarity; viscosities were measured as described and tabulated in Supplementary Information S2. Data were recorded at 490 nm to capture both fast and slow components and the oscillation. Evidently, the ca. 100 fs component and the frequency of the coherent oscillation are independent of solvent viscosity within experimental error (as 132±15 cm^−1^, determined by a Fourier Transform analysis, cf. Figure 3). In contrast both of the slower relaxation times both becomes markedly longer with increasing viscosity (see also Supplementary Information S2). This result accords with the enhanced emission intensity in viscous solvents (Figure 4A).

## 3 Discussion

Ultrafast fluorescence spectroscopy with sufficient time resolution provides insight into the relaxation of the FC excited state. The present data for **I**_c_ shows that the FC state relaxes on the time scale of *ca*. 100 fs. This exceptionally fast relaxation is assigned to the reduction in bond order of the bridging C=C bond upon electronic excitation, which permits the rapid release of steric strain associated with the clash of the two naphthalene units in **I**_c_ (Scheme 1).^10^ The viscosity dependent study (Figure 4) shows that neither the rate nor the amplitude of this ultrafast process are reduced by solvent friction in the range of viscosity studied. Thus the relaxation coordinate associated with FC state decay probably does not displace a large volume of solvent. Similar behavior, a volume conserving relaxation coordinate, was observed in second generation molecular rotary motors, and proposed to involve a pyramidalisation coordinate at a bridging carbon atoms, a conclusion supported by theoretical calculations of excited state dynamics.^6^, ^13^ The present data (Figures 2, 4) indicate that a similar coordinate plays a role in the primary excited state relaxation step of the first generation motors.

The wavelength dependent measurements (Figure 2) show that ultrafast relaxation of the FC state is a consequence of structural relaxation in the **I**_c_ excited state, rather than population decay from S_1_, and leads to a new emissive excited state. We assign the reduced amplitude in the longer lived emission to a lower transition moment associated with the structure populated on the 100 fs timescale. Sension et al. reached the same conclusion in their earlier study of transient absorption and stimulated emission.^8^ From the wavelength dependence measurement (Figure 2) it is evident that the ultrafast decay of the FC state has its main amplitude on the blue side of the emission spectrum. This is consistent with its emission contribution, which decays in ca. 100 fs, having a mirror image relationship with the absorption spectrum (Figure 1). The ultrafast relaxation is to a state with a red‐shifted emission spectrum and a reduced transition dipole moment; it is the latter emission that dominates the steady state fluorescence spectrum, by virtue of its much longer lifetime. The lack of a resolved 100 fs rise time at the longest emission wavelength measured is indicative of the overlap of the picosecond red‐shifted emission with the more strongly allowed 100 fs lifetime blue‐shifted spectrum.

The oscillations superimposed on the picosecond emission (Figure 2) are assigned to a coherently excited low frequency mode in the excited electronic state with a large displacement with respect to the ground state. While calculations for excited state modes in unstable excited states remain challenging, we note that some low frequency modes in the electronic ground state are calculated to reflect a ‘scissoring’ motion of the two ring systems (see Supplementary Information section S3). Such low frequency modes are expected to be displaced following S_0_→S_1_ excitation localised on the bridging C=C bond. It is noteworthy that this excited state vibrational coherence survives structural relaxation from the FC state to the new emissive state. Such structural relaxation along an anharmonic potential energy surface would normally lead to dephasing of the initially excited coherence, suppressing its appearance in the final state. An exception to this is when the new state is formed impulsively from the FC state, i. e. on a time scale that is faster than the inverse frequency of the modes excited in the final state. Such coherent dynamics in product states have been reported in a number of cases, including ultrafast internal conversion, excited state proton transfer and, most relevant to the present data, the formation of internal charge transfer states, which also involves significant nuclear and electronic reorganisation.^14^

Here the new emissive state is formed in ca. 100 fs from the FC state, meaning it is possible to impulsively excite modes up to ca 170 cm^−1^, consistent with our observations of a mode focused around 138 cm^−1^ (Figure 3B).14a As discussed further below, we suggest that the emissive state formed impulsively from FC relaxation is a distorted form of **I**_c_*. Thus, the structural changes in 100 fs are not as extensive as required for the final formation of **I**_t_*, and the sub picosecond dephasing of the impulsively excited modes precedes the major structural change associated with the cis to trans isomerisation. Interestingly the observation of coherences decaying on the hundreds of femtoseconds time scale raises the possibility of coherent control over motor function,^15^ provided that the mode excited is also coupled to the reaction coordinate. Resonant Femtosecond Stimulated Raman Spectroscopy (FSRS) measurements along with excited‐state simulations will enumerate directly the significance of coupled vibrational modes in driving efficient photoisomerisation; such experiments are planned.

The population decay of the relaxed emissive state is non‐single exponential with a wavelength dependent mean lifetime of 5–10 picoseconds (Supporting Information S2). This is significantly longer than was observed for the second generation motors (1–2 ps).^6^, 7b Further, second generation motors had a weaker transition moment in the relaxed state. We interpret the longer lived and brighter state in **I**_c_ as indicating the presence of a barrier along the reaction coordinate which is absent in second generation motors. The effect of the barrier is to trap the excited state population in a more strongly radiative part of the excited state surface. It is counterintuitive that the first‐generation motors should have a barrier in the reaction coordinate yet have a higher yield of product than the second generation motors. This result shows that radiationless decay over the barrier does not lead directly to **I_t_**, but rather to the region of the excited state surface where the conical intersection which funnels population to the ground state is located. It is the position and slope associated with the conical intersection that determines the partitioning between **I_c_** and **I_t_**, on the ground state surface.

A non‐single exponential decay is observed across the entire spectrum, and cannot therefore be associated with a relaxation process within the fluorescent state (such as vibrational cooling or solvation dynamics for example, which mainly contribute to decay on the blue edge). We therefore suggest that the non‐exponential and wavelength dependent decay reflects multiple decay pathways for population decay, i. e. distinct pathways from the fluorescent state to the conical intersection(s), which subsequently transfers population to **I**_t_. Earlier studies of second generation motors have pointed to the existence of multiple minima on the excited state surface and multiple conical interesctions.13a If the excited state of the first generation motors also has such a complex potential landscape this could result in the multimodal relaxation observed.

Finally, the fluorescence decay was studied as a function of viscosity by varying the volume fraction of ethylene glycol in an ethanol:ethylene glycol mixture between 0 and 1. This has the effect of varying the viscosity 15 fold, without changing temperature, maintaining approximately constant polarity and retaining an H‐bonding environment. While the relaxation from the FC state was unaffected, both of the picosecond decay components were observed to be a function of medium viscosity (Supporting Information S2), and the mean lifetime and fluorescence yield increased by factors of 3–4 over the full range of viscosity. This approximately *η* ^1/2^ dependence indicates that the coordinate leading to quenching of the fluorescence is one which requires displacement of solvent molecules. Thus, the radiationless decay coordinate from the emissive state formed in 100 fs from the FC state involves torsion about the connecting C−C bond, a feature which was also observed in the second generation motors.^6^

## 4 Conclusions

Excited state dynamics for the first generation photomolecular motor **I**_c_ involves a 100 fs friction independent decay of the FC state to populate a longer lived red‐shifted emissive state, which itself undergoes non single exponential population decay via a friction dependent coordinate. This two coordinate relaxation has much in common with earlier observations on second generation motors. This similarity suggests an assignment to fast relaxation involving a pyramidalisation coordinate followed by slower friction dependent C−C bond torsion; a more definitive assignment will require a detailed calculation of excited state dynamics. The non‐single exponential decay of the fluorescent state was assigned to a complex excited state potential with multiple decay pathways. Key differences between the two generations of molecular motor are the longer lifetime and higher transition moment of **I**_c_ in the first‐generation motors. This is ascribed to the existence of a barrier in **I**_c_ along the excited state relaxation coordinate which traps population for longer on a more strongly radiative part of the potential energy surface. It is interesting to note that the first‐generation motor has the higher quantum yield for isomerisation even though it has the longer excited state lifetime. This indicates that the radiationless decay is not directly to **I**_t_, but rather to a ground or dark state from which population partitions between **I**_t_ and the original ground state. A more complete picture of the structure‐dynamics correlation of lifetime and isomerisation quantum yield will aid the design and the synthesis of more efficient overcrowded‐alkene based molecular motors.

## Supporting information

As a service to our authors and readers, this journal provides supporting information supplied by the authors. Such materials are peer reviewed and may be re‐organized for online delivery, but are not copy‐edited or typeset. Technical support issues arising from supporting information (other than missing files) should be addressed to the authors.

Supplementary
