# Supplementary material for: Ultrafast Excited State Dynamics in a First Generation Photomolecular Motor
Source: Chemphyschem. 2020 Feb 3;21(7):594–9. doi: 10.1002/cphc.201901179 (PMC7187380; doi:10.1002/cphc.201901179)
Supplement: Supplementary file 1 — Supplementary [file CPHC-21-594-s001.pdf]

CHEMPHYSCHEM

Supporting Information

Ultrafast Excited State Dynamics in a First Generation Photomolecular Motor

Andy S. Sardjan, Palas Roy, Wojciech Danowski, Giovanni Bressan, Laura Nunes dos Santos Comprido, Wesley R. Browne, Ben. L. Feringa,* and Stephen R. Meech*© 2020 The Authors. Published by Wiley-VCH Verlag GmbH & Co. KGaA. This is an open access article under the terms of the Creative Commons Attribution License, which permits use, distribution and reproduction in any medium, provided the original work is properly cited.An invited contribution to a Special Collection in Honor of O. Poizat

SUPPLEMENTARY INFORMATION

S1 Further experimental details

Single shot fluorescence spectra

Flow and conversion calculations

Raman Spectroscopy

S2 Data Tables

Wavelength dependent decay data

Viscosity dependent decay data

Viscosities

S3 Computational Details

Coordinates, Raman spectrum and characteristic low frequency modes.

S1. Further experimental details

S1a Single Shot Steady State Fluorescence

One of the difficulties in obtaining the emission spectrum of $\mathbf{I}_c$ is that it photoconverts during collection. To circumvent this we used a 1 Hz pulsed laser to excite fluorescence in a flow system. Solutions of $\mathbf{I}_c$ in ethanol and ethylene glycol were prepared (at an absorbance of 0.2 at 355 nm). These two isoabsorptive solutions were used to prepare solutions with a range of solvent volume fractions. (100:0, 75:25, 50:50, 25:75 and 0:100 ethanol:ethylene glycol). The absorption spectra of each solution were recorded (specord 600) to confirm the absorbance at 355 nm. The third harmonic of a nanosecond Nd:YAG laser (Innolas spitlight 200) was used for excitation, with fluorescence spectra recorded under conditions where conversion to the metastable form is minimal. The pulse power was kept below 1.0 mJ and the solution was flowed through the beam using a flow cell and a syringe pump to avoid build-up of the metastable isomer during the experiment. The flow rate was sufficient to ensure a fresh solution was present for each pulse (1 Hz). This data is shown in Figures 1 and 4.

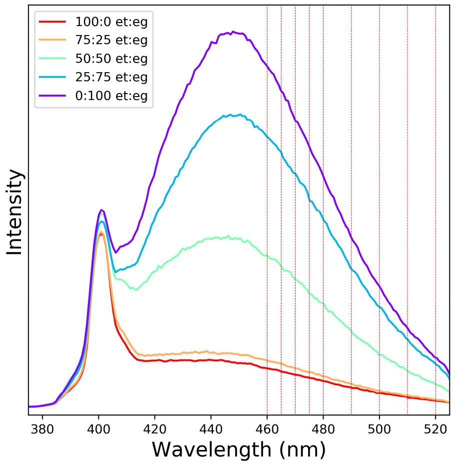

Figure S1. Fluorescence spectra of cis motor over a range of volume fractions of ethanol and ethylene glycol. Intensity is corrected for differences in absorbance. Vertical lines correspond to wavelengths where fluorescence upconversion measurements were made (see Figure 2).

S1b Flow and Conversion Calculations

A second difficulty is ensuring that the solute $\mathbf{I}_c$ is not converted in the beam during the quasi CW up-conversion measurement. It is necessary to ensure that conversion is not so high that the probability of photoproduct absorption (and subsequent emission) becomes significant. Here we calculate the flow rate and conversion rate under our conditions.

Using a graduated cylinder, ethanol and ethylene glycol were pumped through the cell using different speed settings of the pump. The time was measured until 40 mL of solvent was pumped out. The results are given in Figure 1.

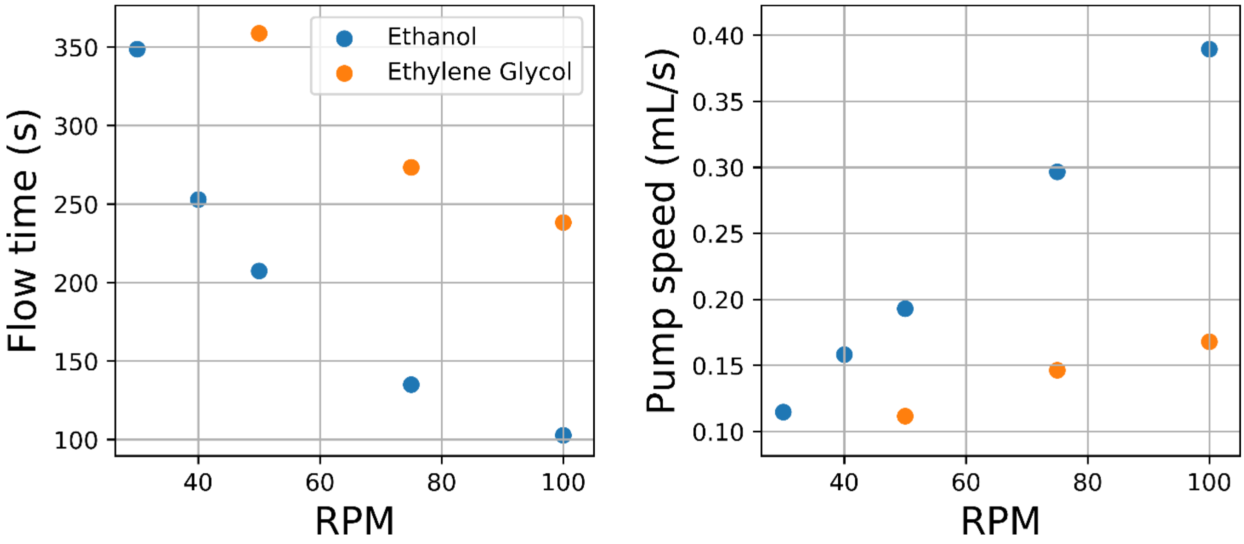

Figure S2: Measured flow times and pump speeds

Conversion in the confocal volume

In order to see if we would get significant conversion in the upconversion experiment, the following calculation was done.

A 1 mm pathlength cuvette was used, and assuming the beam is focused to a cylinder of length 1 mm and radius 25 μm, we calculated the focal volume to be:

$V = \pi r^2 l_{path}$ (1)

Where r is the radius of the cylinder and l the height. We use a concentration such that the absorbance is around 0.5. The molar absorptivity of the $\mathbf{I}_c$ isomer at 400 nm is around 4000 M^{-1} cm^{-1}. Using the Beer-Lambert law:

$A = \varepsilon c l = -\log(T)$ (2)

Where ε is the molar absorptivity, c the concentration of sample and l the path length of the cell. T is the percent transmittance. With this equation we can calculate the desired concentration of the sample and also the amount of light that is absorbed by the sample in percentage:

$T = 10^{-A}$ (3)

We can use equation (4) to get the percentage of light absorbed, which is one minus the percentage of light transmitted:

$\%A = 1 - T = 1 - 10^{-A}$ (4)

Using the measured flow rate (f) of 0.39 mL/s and the estimated size of the confocal volume, we can determine the speed of the molecules through the confocal volume (neglecting diffusion).

$f = A_{cross}v$ (5)

Where A_{cross} is the cross section area and v the speed of the molecules through the confocal volume. In our case A_{cross} is a rectangle of 50 μm by 1 mm. Using this speed we can determine the time a molecule would reside in the confocal volume (t_R), if it passes through the center then l = 50 µm):

$$t = \frac{l}{v} = \frac{lA}{f} = \frac{50\ \mu m * 1\ mm * 50\ \mu m}{0.39\ mL/s} = 1.67 \times 10^{-5} s \quad (6)$$

Furthermore, since we measure the power of the 400 nm beam hitting the sample and, using the concentration and volume we can determine the number of molecules in the confocal volume, we can thus determine the number of conversions per second. First, we start by calculating the number of photons hitting the confocal volume per second. Knowing the power of the beam P = 5 mW and the wavelength λ = 400 nm, we can calculate the number of photons per second hitting the confocal volume R:

$$R = \frac{P}{E} = \frac{P\lambda}{hc} \quad (7)$$

From equation (4), we know the percentage of light absorbed. Using this and multiplying by the quantum yield of conversion, φ we can calculate the number of molecules converting per second:

$$conv = \phi R.\%A = \phi \frac{P\lambda}{hc}(1 - 10^{-A}) \quad (8)$$

Using this initial rate, we can calculate the minimal time it takes to absorb enough photons to convert the entire confocal volume. For this we need the number of molecules in the confocal volume, which is the concentration times the volume, the concentration can be calculated from the absorbance and the volume from equation (1), $c = A/(\epsilon l_{path})$:

$$N_{confocal} = c * V * N_A = \frac{A}{\epsilon l_{path}} \pi r^2 l_{path} N_A = \frac{A}{\epsilon} \pi r^2 N_A \quad (9)$$

Since the concentration from the Beer-Lambert law would be in molar units, we need to convert to number of molecules. Therefore, the time it takes to absorb enough photons to convert the entire confocal volume is given by:

$$t_{convert} = \frac{N_{confocal}}{conv} = \frac{\frac{A}{\epsilon}\pi r^2 N_A}{\phi \frac{P\lambda}{hc}(1-10^{-A})} = \frac{hc\pi r^2 N_A}{\phi P \lambda \epsilon} \frac{A}{(1-10^{-A})} \quad (10)$$

Substituting all the relevant values gives:

$t_{convert} = 2.5 \times 10^{-4}$ s (11)

The actual conversion time is longer since the concentration decreases when molecules are converted. The conversion time is longer than the residence time, so conversion should not be an issue on the timescale of the experiment at the powers employed.

S1c Raman spectroscopy

Raman spectra in the solid state and in solution were obtained using a Raman microscope at 785 nm and macrosampling unit (Perkin Elmer Raman station), respectively. The spectra were compared with the calculated spectrum. Overall there is the expected close correspondence between the spectra, however minor difference in band position are apparent between the solid state and solution spectra. Notably the spectra are similar in the low wavenumber region (90 to 200 cm^{-1}) indicating that the bands observed in the solid are not due to phonons. A full list of band positions are given at the end of the SI. The displacements associated with calculated low wavenumber modes are also shown below (Figure S5) and indicate that most of the modes in this region correspond to essentially similar flexing of the entire molecule.

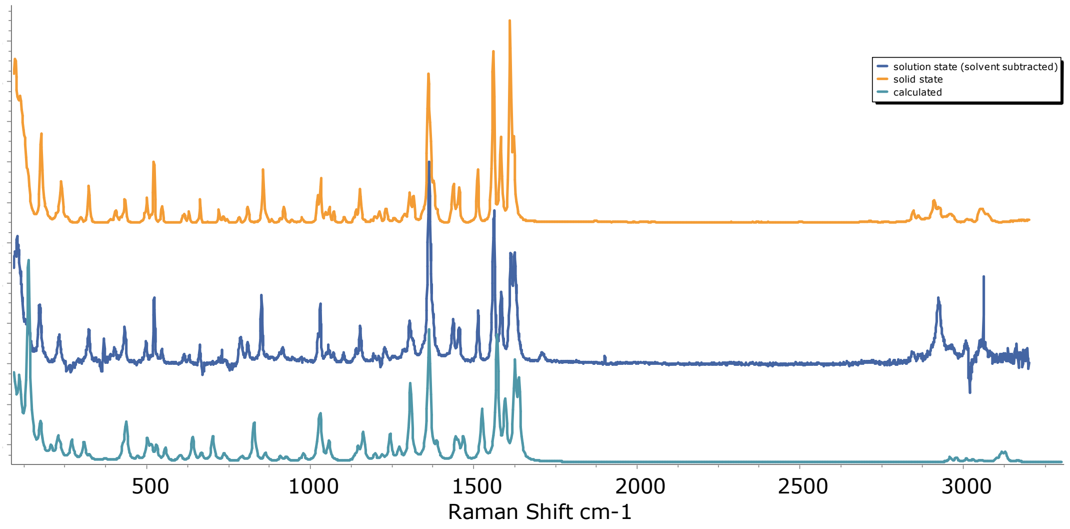

Figure S3. Raman spectrum at 785 nm of ***I$_c$*** *in the solid state (orange), in $CHCl_3$ (blue) Contributions from solvent were subtracted. Negative signals are due to imperfect solvent subtraction. The calculated spectrum (scaling factor of 0.98 applied) in green.*

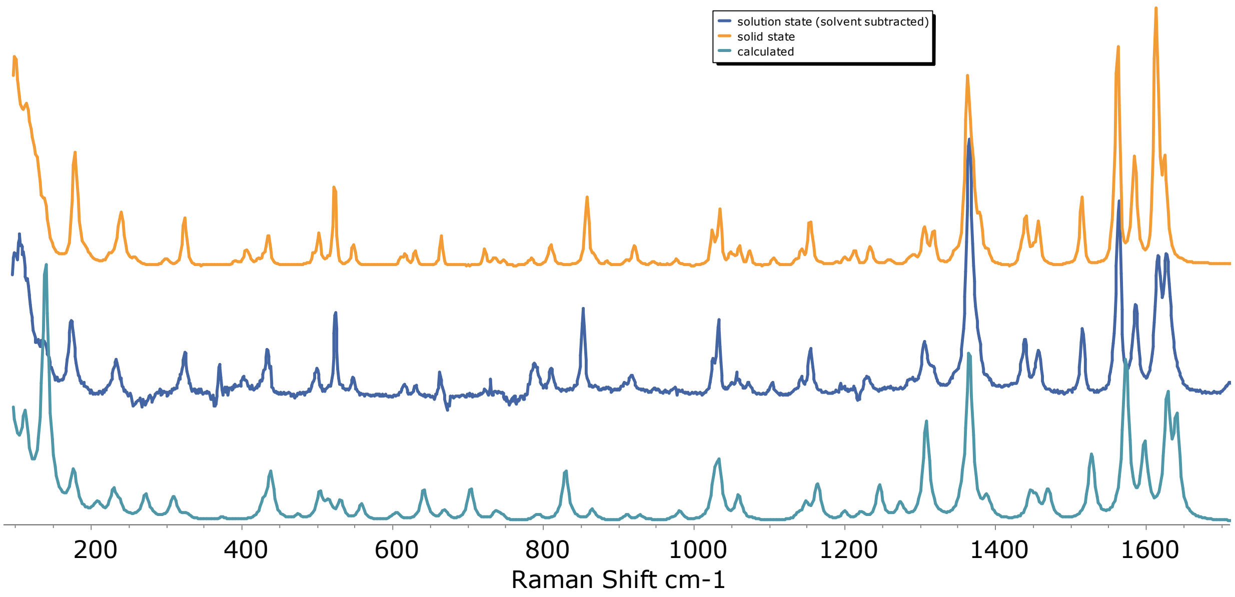

*Figure S4. Fingerprint region of the Raman spectrum at 785 nm of **I**$_c$ in the solid state (orange), in $CHCl_3$ (blue) Contributions from solvent were subtracted. Negative signals are due to imperfect solvent subtraction. The calculated spectrum (scaling factor of 0.98 applied) in green.*

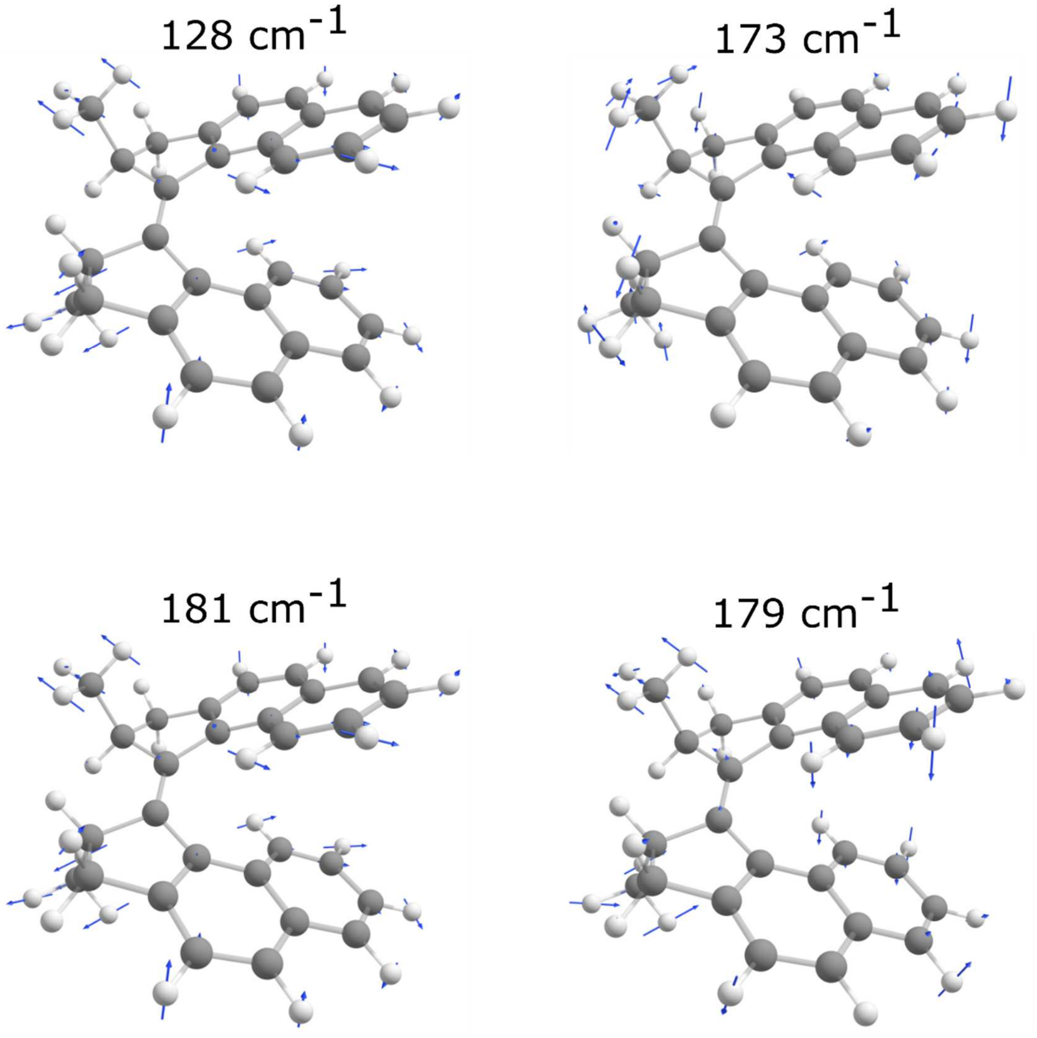

Figure S5 Computed modes at indicated wavenumbers indicating delocalized nature.

S2 Data Tables

Table S1 Biexponential fit parameters for **I**$_c$ fluorescence decay measured as a function of emission wavelength. Data are fit after the initial 100 fs decay of the FC state has completed.

Wavelength (nm)	460	465	470	475	480	490	500	510	520
A_1	0.68	0.66	0.6	0.64	0.61	0.58	0.59	0.62	0.51
τ_1 (fs)	600	576	752	575	585	687	671	1043	728
A_2	0.32	0.34	0.4	0.36	0.39	0.42	0.41	0.38	0.49
τ_2 (fs)	10696	10066	9575	8367	7327	7051	6244	7344	5446
$<\tau>$ (fs)	9602	9130	8641	7503	6588	6285	5500	6150	4864

The average lifetime was calculated using:

$$< \tau >= \frac{A_1\,\tau_1^2 + A_2\,\tau_2^2}{A_1\,\tau_1 + A_2\,\tau_2}$$

Table S2 Biexponential fit parameters at 490 nm for **I**$_c$ fluorescence decay as a function of EtOH:EG composition.

EtOH:EG	100:0	75:25	50:50	25:75	0:100
A_1	0.58	0.6	0.48	0.56	0.18
τ_1 (fs)	687	1048	925	3068	1616
A_2	0.42	0.4	0.52	0.44	0.82
τ_2 (fs)	7051	10682	9618	22216	17267
$<\tau>$ (fs)	6285	9430	8901	19332	16957

Table S3 Viscosity data determined using an Ubbeholde viscometer at 293 K

EtOH:EG	ρ (g L^{-1})	η (mPa s)
100:0	786.638	2.019
75:25	877.165	3.574
50:50	959.746	6.101
25:75	1035.366	10.131
0:100	1108.374	17.238

S3 Computational Details

Calculations were carried out using the electronic structure code Gaussian.[1] Geometries were optimized at the B3LYP[2]-D3(BJ)[3]/def2-TZVP[4] level of theory with an ultrafine grid and tight convergence. Raman intensities were also calculated at this level of theory at 298.15 K.

XYZ Coordinates

C	2.662544000	2.412818000	-1.109599000	$E_{B3LYP\text{-}D3(BJ)/def2\text{-}TZVP}$ = -1081.97391371
C	-0.674342000	1.643206000	0.019371000	
C	-1.657374000	0.549194000	0.025783000	
C	-2.791950000	0.968876000	0.704269000	
C	-2.662543000	2.412818000	1.109600000	
C	-1.493538000	2.917273000	0.225810000	
C	0.674342000	1.643206000	-0.019371000	
C	1.657374000	0.549194000	-0.025783000	
C	2.791951000	0.968876000	-0.704269000	
C	1.493538000	2.917272000	-0.225810000	
C	3.870803000	0.103685000	-0.934879000	
C	1.631810000	-0.733285000	0.592142000	
C	-1.631810000	-0.733285000	-0.592141000	
H	3.584859000	2.975941000	-0.952157000	
H	2.403945000	2.499538000	-2.170213000	
C	2.722539000	-1.621130000	0.330344000	
C	3.817153000	-1.180197000	-0.452161000	
C	-3.870803000	0.103686000	0.934879000	
C	-3.817153000	-1.180196000	0.452161000	
C	-2.722539000	-1.621129000	-0.330344000	
C	-2.699060000	-2.920420000	-0.890799000	
C	-0.621327000	-1.157536000	-1.484125000	
C	-1.674950000	-3.317253000	-1.709242000	
C	-0.639120000	-2.416075000	-2.026045000	
C	2.007898000	3.493944000	1.097014000	
C	-2.007898000	3.493944000	-1.097014000	
H	4.734795000	0.446852000	-1.490835000	
H	4.633227000	-1.868909000	-0.634083000	
H	0.173622000	-0.474791000	-1.738696000	
H	0.149393000	-2.720999000	-2.701739000	
H	-1.668934000	-4.314151000	-2.130718000	
H	-3.518106000	-3.594337000	-0.669986000	
H	-4.633227000	-1.868908000	0.634083000	
H	-4.734794000	0.446853000	1.490835000	
H	2.617829000	4.382863000	0.921073000	
H	1.182170000	3.772136000	1.753338000	
H	2.619614000	2.758784000	1.623516000	
H	-2.619614000	2.758785000	-1.623516000	
H	-1.182169000	3.772137000	-1.753338000	
H	-2.617829000	4.382863000	-0.921073000	
H	-2.403944000	2.499538000	2.170213000	
H	-3.584858000	2.975941000	0.952157000	
H	-0.914379000	3.678459000	0.746353000	
H	0.914379000	3.678459000	-0.746354000	
C	2.699060000	-2.920420000	0.890799000	
C	0.621327000	-1.157535000	1.484126000	
C	0.639120000	-2.416075000	2.026045000	
C	1.674949000	-3.317253000	1.709242000	
H	-0.173622000	-0.474791000	1.738697000	
H	-0.149393000	-2.720998000	2.701739000	
H	1.668933000	-4.314151000	2.130718000	
H	3.518105000	-3.594337000	0.669986000	

Table S4 Peak position and relative intensities for spectra (Figure 3) in solid state, solution and calculated.

Raman shift [1/cm]	solid state (rel. Intensity)	Raman shift [1/cm]	solution state (rel. Intensity)	Raman shift [1/cm]	calculated (rel. Intensity)
177.81	0.4357	104.2	0.6184	35.9242	1
239.3	0.2013	172.97	0.2952	43.9189	0.82499
299.07	0.0263	232.76	0.1419	55.2154	0.110062
323.59	0.1781	324.38	0.1706	81.8538	0.051599
405.68	0.0606	370	0.1252	82.5028	0.248164
435.37	0.1133	401.74	0.0796	105.6354	0.039687
501.5	0.1177	433.88	0.1820	123.7437	0.040035
523.29	0.2906	499.22	0.1086	132.7901	0.039121
547.25	0.0749	523.44	0.3207	162.968	0.059854
629.49	0.0530	546.8	0.0720	173.8385	0.036245
664.26	0.1095	615.52	0.0478	178.5872	0.007334
721.98	0.0640	630.28	0.0400	222.5964	0.03822
784.73	0.0258	662.64	0.0857	233.961	0.008573
809.46	0.0752	729.74	0.0620	251.1505	0.012972
856.14	0.2301	788.04	0.1313	294.8046	0.001755
918.91	0.0644	809.93	0.1090	316.0748	0.004551
975.88	0.0238	852.26	0.3383	327.2351	0.029057
1033.4	0.2077	917.34	0.0819	393.2534	0.001182
1059.5	0.0734	1031.9	0.2964	402.7384	0.010633
1105.1	0.0275	1056.2	0.0895	429.8721	0.005486
1153.1	0.1660	1071.9	0.0575	434.7406	0.00668
1212.6	0.0567	1103.3	0.0540	439.8508	0.026862
1232.9	0.0698	1153.9	0.1874	496.8108	0.001395
1256.4	0.0203	1194.2	0.0498	504.3255	0.007844
1305.5	0.1457	1229.8	0.0760	514.1482	0.012584
1362.9	0.7155	1305.4	0.2123	523.8587	0.002229
1439.4	0.1868	1364.8	0.9954	533.3569	0.04454
1513.8	0.2589	1438.4	0.2232	545.0552	0.002086
1561.6	0.8268	1456.6	0.1812	559.0915	0.008792
1584.1	0.4196	1514.9	0.2628	628.443	0.007451
1612.4	0.9761	1563	0.7580	629.012	0.004225
		1585.4	0.3557	648.8838	0.005148
		1615.4	0.5463	678.2897	0.010422
		1626.4	0.5526	744.6389	0.003892
		1710.4	0.0575	748.5332	0.001529

						750.11	0.001747
						756.4224	0.002603
						797.3976	0.003124
						802.9735	0.001291
						820.0367	0.012012
						860.0513	0.009588
						864.0958	0.043457
						887.3937	0.001807
						899.2811	0.001491
						920.1665	0.001257
						929.0738	0.00817
						930.6405	0.00322
						990.3265	0.007091
						1042.655	0.036065
						1049.962	0.00335
						1053.289	0.028164
						1066.648	0.004043
						1072.498	0.007134
						1073.185	0.017532
						1095.222	0.001605
						1096.306	0.003384
						1121.364	0.007697
						1154.999	0.003946
						1169.59	0.004624
						1169.753	0.00707
						1178.318	0.018407
						1181.679	0.001168
						1182.146	0.015881
						1188.461	0.00167
						1211.608	0.001161
						1221.241	0.007318
						1238.728	0.001406
						1240.087	0.013332
						1257.006	0.018688
						1283.767	0.006283
						1292.704	0.001372
						1312.144	0.019095
						1335.806	0.00294
						1337.029	0.056565
						1349.424	0.001389
						1354.665	0.013467
						1375.151	0.003322
						1375.594	0.006666

						1385.753	0.128261
						1389.144	0.035671
						1400.617	0.038002
						1409.194	0.003401
						1412.841	0.013115
						1469.417	0.017683
						1470.253	0.008315
						1482.5	0.007799
						1483.612	0.013053
						1494.443	0.023916
						1495.059	0.001732
						1497.686	0.003298
						1498.106	0.001542
						1499.498	0.006299
						1552.834	0.058747
						1554.622	0.010687
						1597.3	0.17029
						1600.89	0.03881
						1623.659	0.093093
						1626.644	0.021728
						1652.772	0.177803
						1664.334	0.102739

References

[1] Gaussian 16, Revision B.01, M. J. Frisch, G. W. Trucks, H. B. Schlegel, G. E. Scuseria, M. A. Robb, J. R. Cheeseman, G. Scalmani, V. Barone, G. A. Petersson, H. Nakatsuji, X. Li, M. Caricato, A. V. Marenich, J. Bloino, B. G. Janesko, R. Gomperts, B. Mennucci, H. P. Hratchian, J. V. Ortiz, A. F. Izmaylov, J. L. Sonnenberg, D. Williams-Young, F. Ding, F. Lipparini, F. Egidi, J. Goings, B. Peng, A. Petrone, T. Henderson, D. Ranasinghe, V. G. Zakrzewski, J. Gao, N. Rega, G. Zheng, W. Liang, M. Hada, M. Ehara, K. Toyota, R. Fukuda, J. Hasegawa, M. Ishida, T. Nakajima, Y. Honda, O. Kitao, H. Nakai, T. Vreven, K. Throssell, J. A. Montgomery, Jr., J. E. Peralta, F. Ogliaro, M. J. Bearpark, J. J. Heyd, E. N. Brothers, K. N. Kudin, V. N. Staroverov, T. A. Keith, R. Kobayashi, J. Normand, K. Raghavachari, A. P. Rendell, J. C. Burant, S. S. Iyengar, J. Tomasi, M. Cossi, J. M. Millam, M. Klene, C. Adamo, R. Cammi, J. W. Ochterski, R. L. Martin, K. Morokuma, O. Farkas, J. B. Foresman, and D. J. Fox, Gaussian, Inc., Wallingford CT, 2016.

[2] (a) A. D. Becke, *Phys. Rev. A* **1988**, *38*, 3098-3100; (b) A. D. Becke, *J. Chem. Phys.* **1993**, *98*, 5648-5652; (c) C. Lee, W. Yang, R. G. Parr, *Phys. Rev. B* **1988**, *37*, 785-789; (d) P. J. Stephens, F. J. Devlin, C. F. Chabalowski, M. J. Frisch, *J. Phys. Chem.* **1994**, *98*, 11623-11627.

[3] (a) S. Grimme, J. Antony, S. Ehrlich, H. Krieg, *J. Chem. Phys.* **2010**, *132*, 154104; (b) S. Grimme, S. Ehrlich, L. Goerigk, *J. Comput. Chem.* **2011**, *32*, 1456-1465.

[4] F. Weigend, R. Ahlrichs, *Phys. Chem. Chem. Phys.* **2005**, *7*, 3297-3305.
